# BioAfrica's HIV-1 Proteomics Resource: Combining protein data with bioinformatics tools

**DOI:** 10.1186/1742-4690-2-18

**Published:** 2005-03-09

**Authors:** Ryan S Doherty, Tulio De Oliveira, Chris Seebregts, Sivapragashini Danaviah, Michelle Gordon, Sharon Cassol

**Affiliations:** 1 Molecular Virology and Bioinformatics Unit, Africa Centre for Health and Population Studies, Doris Duke Medical Research Institute, Nelson R. Mandela School of Medicine, University of KwaZulu-Natal, Durban, South Africa; 2 Biomedical Informatics Research Division, South African Medical Research Council, Cape Town, South Africa; 3 Department of Medical Virology, University of Pretoria, Pretoria, South Africa

## Abstract

Most Internet online resources for investigating HIV biology contain either bioinformatics tools, protein information or sequence data. The objective of this study was to develop a comprehensive online proteomics resource that integrates bioinformatics with the latest information on HIV-1 protein structure, gene expression, post-transcriptional/post-translational modification, functional activity, and protein-macromolecule interactions. The BioAfrica HIV-1 Proteomics Resource  is a website that contains detailed information about the HIV-1 proteome and protease cleavage sites, as well as data-mining tools that can be used to manipulate and query protein sequence data, a BLAST tool for initiating structural analyses of HIV-1 proteins, and a proteomics tools directory. The Proteome section contains extensive data on each of 19 HIV-1 proteins, including their functional properties, a sample analysis of HIV-1_HXB2_, structural models and links to other online resources. The HIV-1 Protease Cleavage Sites section provides information on the position, subtype variation and genetic evolution of Gag, Gag-Pol and Nef cleavage sites. The HIV-1 Protein Data-mining Tool includes a set of 27 group M (subtypes A through K) reference sequences that can be used to assess the influence of genetic variation on immunological and functional domains of the protein. The BLAST Structure Tool identifies proteins with similar, experimentally determined topologies, and the Tools Directory provides a categorized list of websites and relevant software programs. This combined database and software repository is designed to facilitate the capture, retrieval and analysis of HIV-1 protein data, and to convert it into clinically useful information relating to the pathogenesis, transmission and therapeutic response of different HIV-1 variants. The HIV-1 Proteomics Resource is readily accessible through the BioAfrica website at:

## Background

Although the HIV-1 genome contains only 9 genes, it is capable of generating more than 19 gene products. These products can be divided into three major categories: structural and enzymatic (Gag, Pol, Env); immediate-early regulatory (Tat, Rev and Nef), and late regulatory (Vif, Vpu, Vpr) proteins. Tat, Rev and Nef are synthesized from small multiply-spliced mRNAs; Env, Vif, Vpu and Vpr are generated from singly-spliced mRNAs, the Gag and Gag-Pol precursor polyproteins are synthesized from full-length mRNA. The matrix (p17), capsid (p24) and nucleocapsid (p7) proteins are produced by protease cleavage of Gag and Gag-Pol, a fusion protein derived by ribosomal frame-shifting. Cleavage of Nef generates two different protein isoforms; one myristylated, the other non-myristylated. The viral enzymes (protease, reverse transcriptase, RNase H and integrase) are formed by protease cleavage of Gag-Pol. Alternative splicing, together with co-translational and post-translational modification, leads to additional protein variability [1].

**Figure 1:**
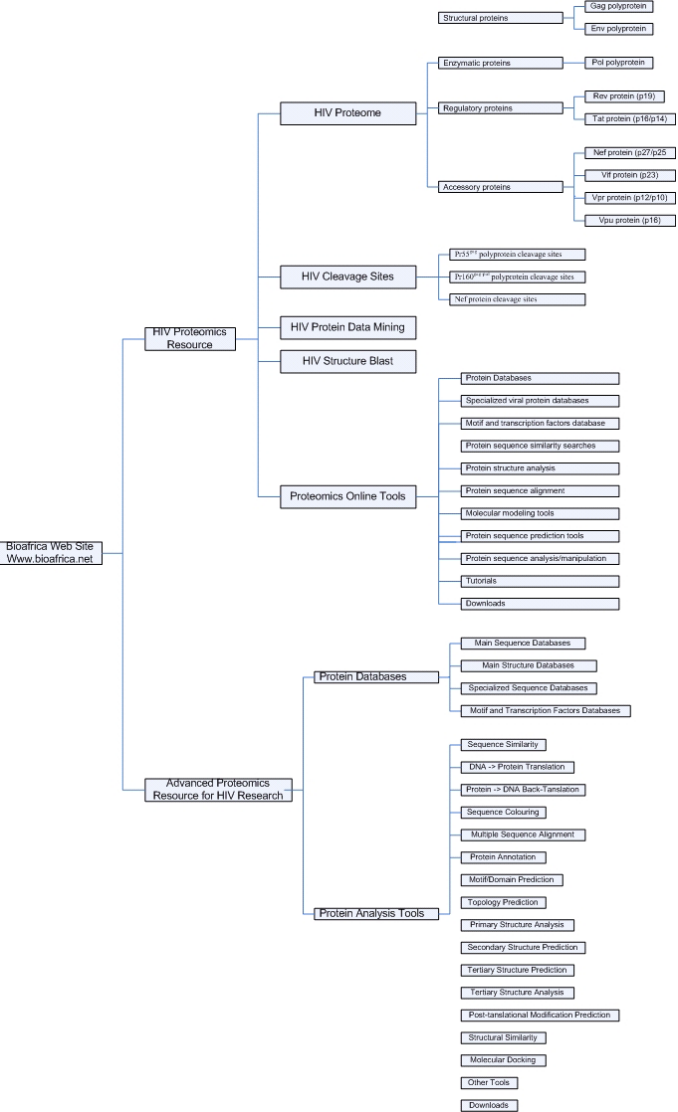
Site map of BioAfrica's HIV-1 Proteomics Resource, showing the separation of Beginner's and the Advanced area of the website, along with all major subject headings.

Phylogenetic analysis, on its own, provides little information about the conformational, immunological and functional properties of HIV-1 proteins, but instead, focuses on the evolution and historical significance of sequence variants. To understand the clinical significance of genetic variation, sequence analysis needs to be combined with methods that assess change in the structural and biological properties of HIV-1 proteins. At present, information and tools for the systematic analysis of HIV-1 proteins are limited, and are scattered across a wide-range of online resources [2,3]. To facilitate studies of the biological consequences of genetic variation, we have developed an integrated, user-friendly proteomics resource that integrates common approaches to HIV-1 protein analysis (Figure 1). We are currently using this resource to better understand the structure-function relationships underlying the emergence of antiretroviral drug resistance, and to examine the process of immune escape from cytotoxic T-lymphocytes (CTLs).

**Figure 2:**
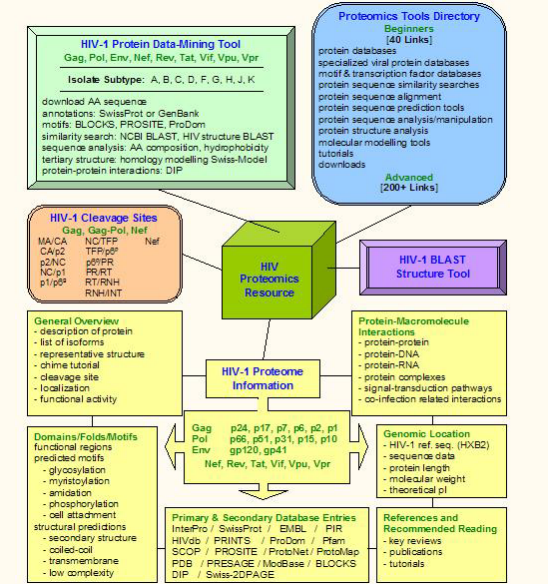
Schematic representation of BioAfrica's HIV-1 Proteomics Resource, showing its five major components: the HIV-1 Proteome (General Overview, Domains/Folds/Motifs, Genomic Location, Protein-Macromolecule Interactions, Primary and Secondary Database Entries, and References and Recommended Readings), the HIV-1 Protease Cleavage Sites section, the HIV-1 Protein Data-mining Tool, the HIV-1 BLAST Structure Tool, and the Proteomics Tools Directory (for Beginners and Advanced investigators).

**Figure 3:**
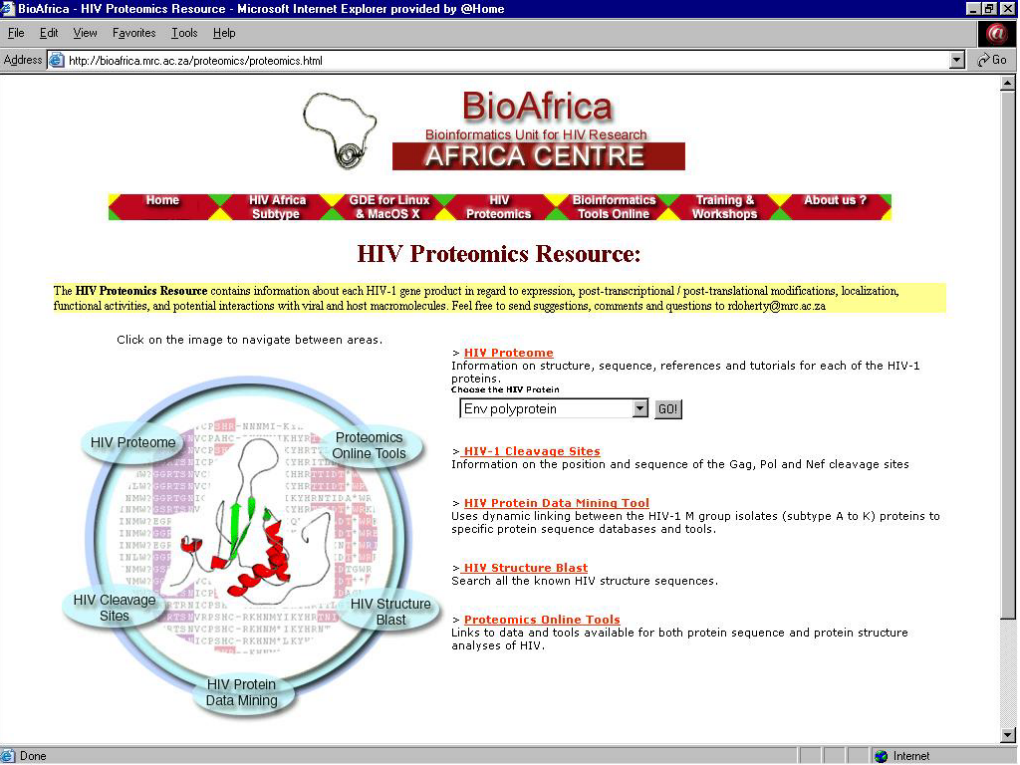
The central webpage of BioAfrica's HIV Proteomics Resource

**Figure 4:**
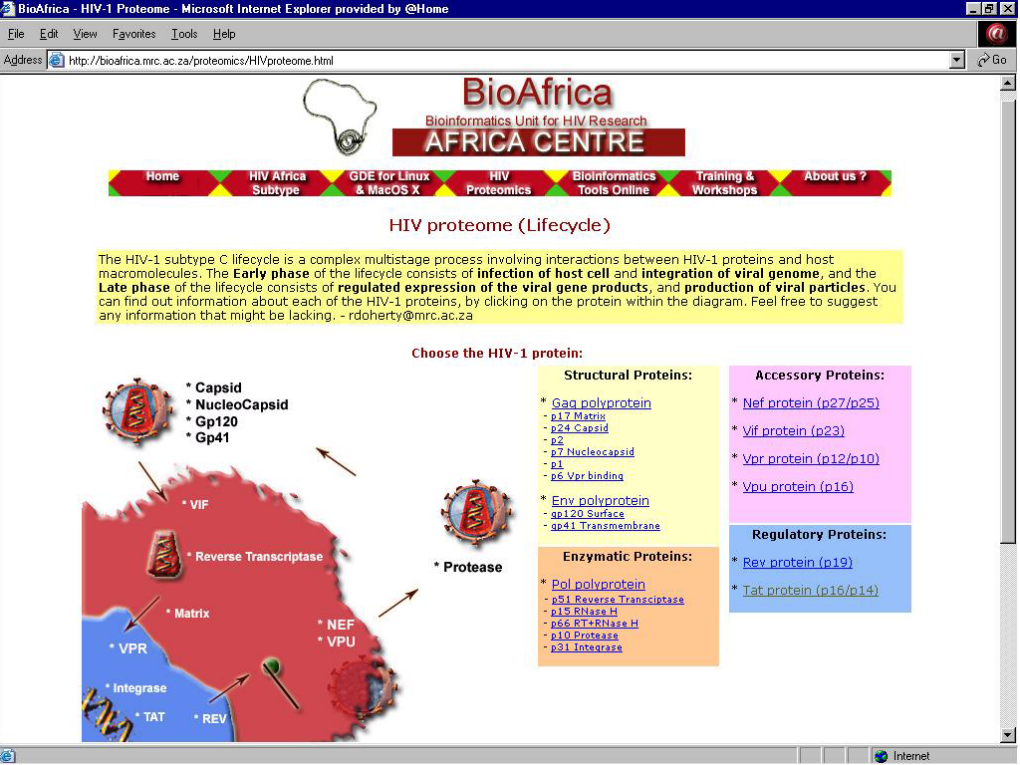
The central webpage of the HIV-1 Proteome section of the BioAfrica website .

**Figure 5:**
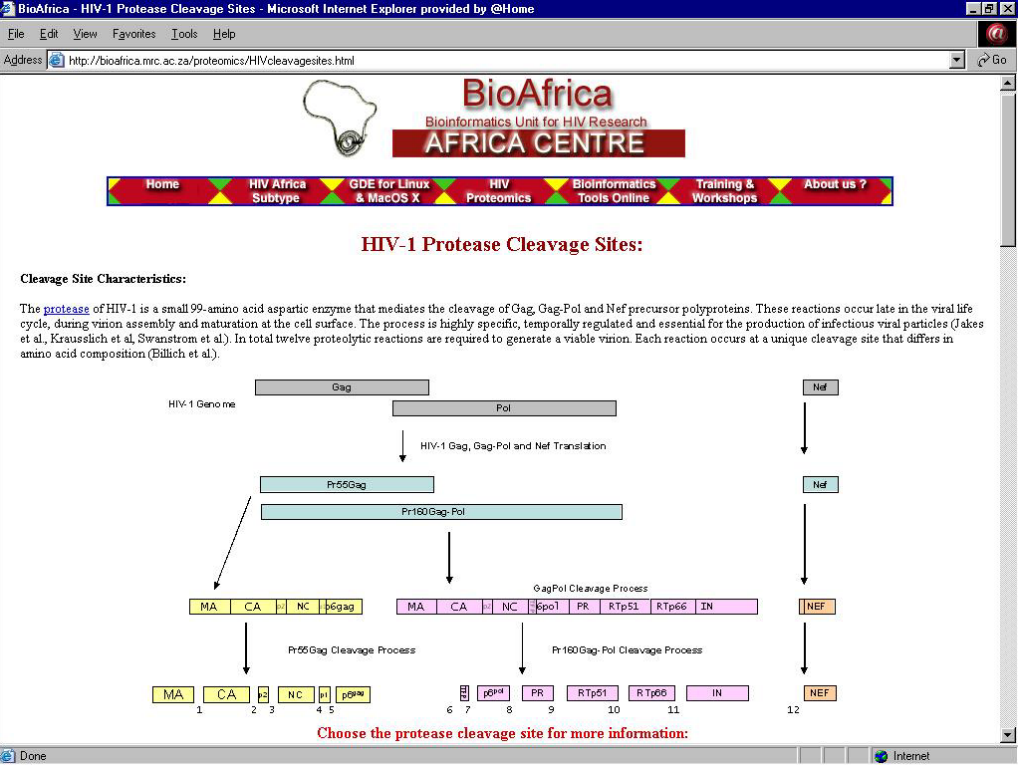
The HIV-1 Protease Cleavage Sites section of the BioAfrica website .

**Figure 6:**
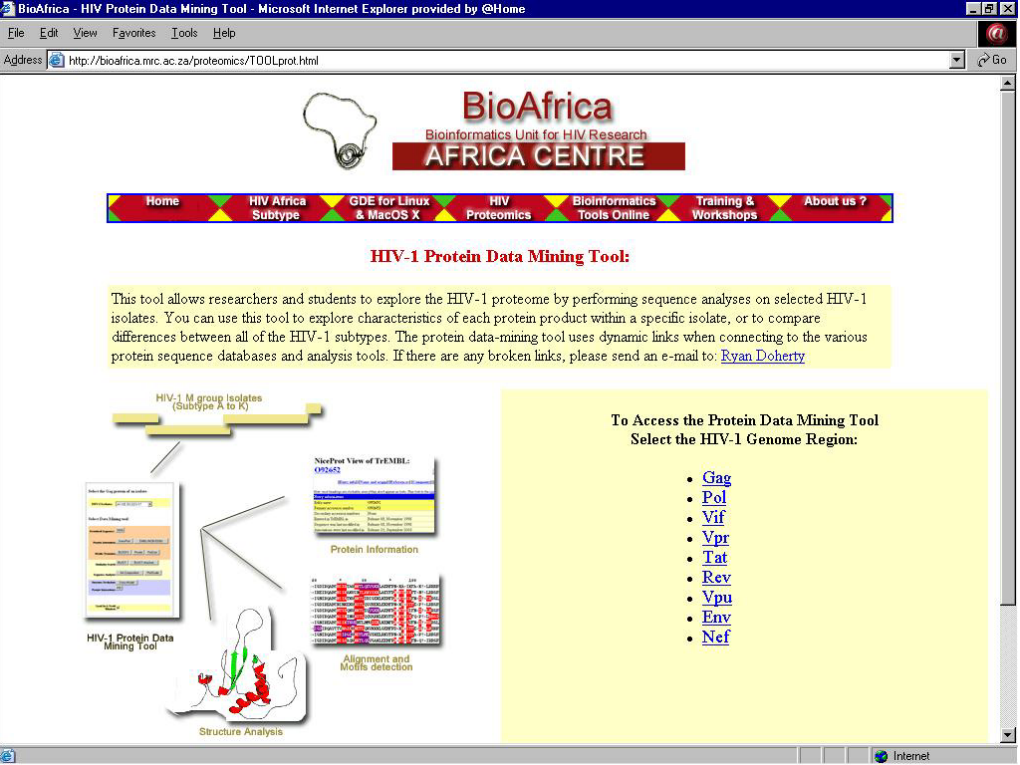
The central webpage of the HIV-1 Protein Data Mining Tool section of the BioAfrica website, where a specific HIV-1 genomic region is selected to be analyzed .

**Figure 7:**
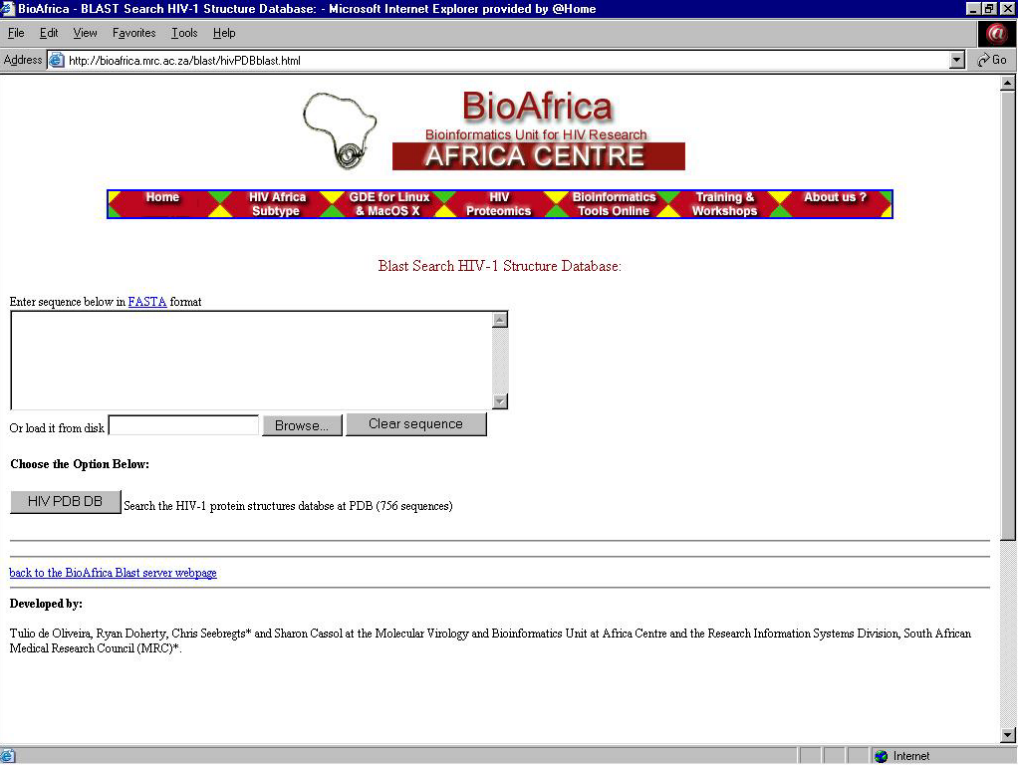
The BLAST HIV-1 protein structure similarity search is an online tool that searches for all protein structure data within the PDB that have an amino acid sequence similar to the query sequence .

**Figure 8:**
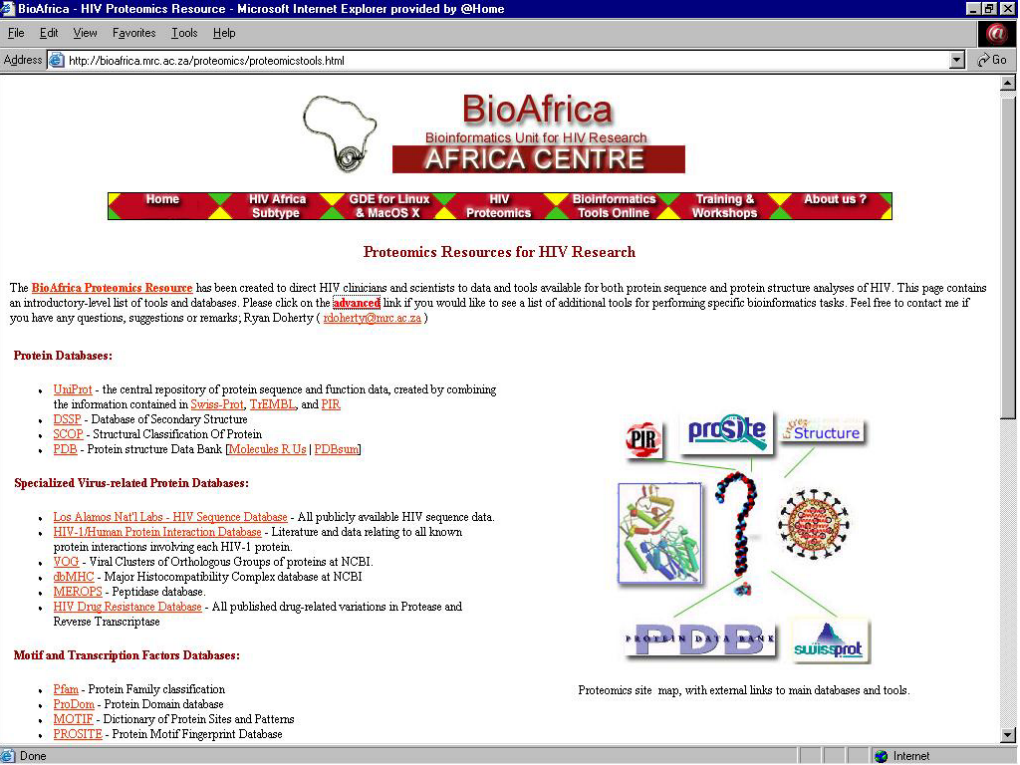
The introductory listing of proteomics resources for HIV research chosen to give a general overview of online tools and databases relevant for the analysis of HIV protein data .

**Figure 9:**
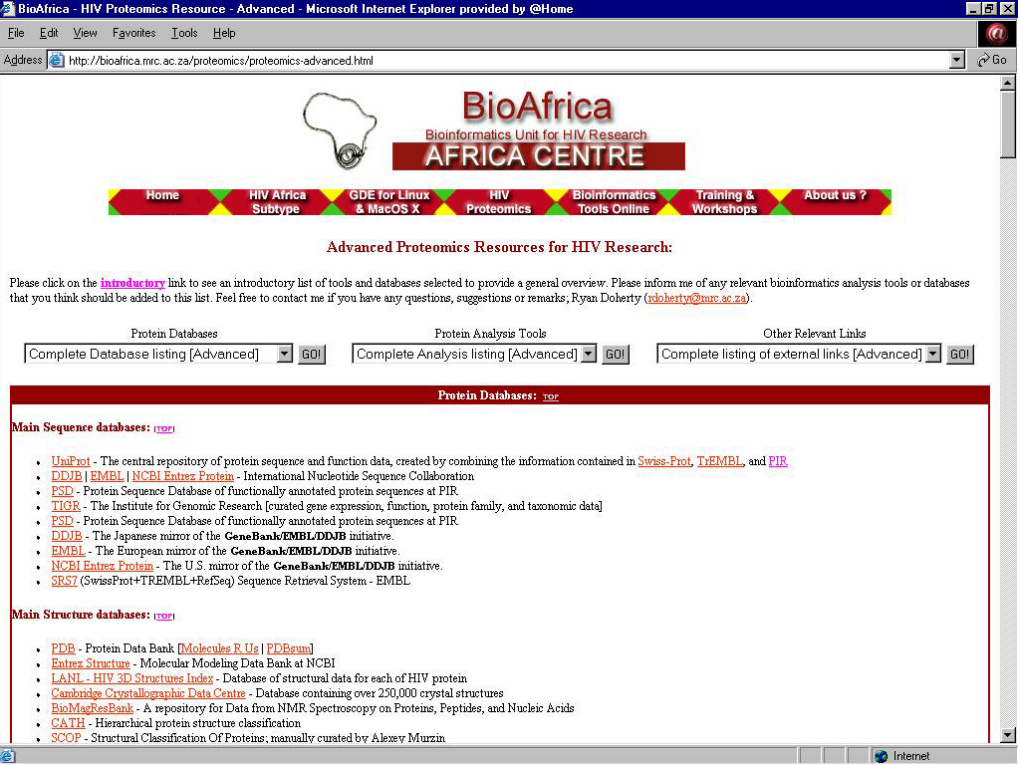
The advanced listing of online tools and databases relevant for the analysis of HIV protein data .

We have categorized the Proteomics Resource into the following main subject headings (Figure 2 &3):

1. ***HIV Proteome ***– Information about structure and sequence, as well as references and tutorials, for each of the HIV-1 proteins (Figure 4);

2. ***HIV-1 Cleavage Sites ***– Information about the position and sequence of HIV-1 Gag, Pol and Nef cleavage sites (Figure 5);

3. ***HIV Protein Data Mining Tool ***– Application for detecting the characteristics of HIV-1 M group isolate (subtype A to K) proteins using information available in public databases and tools (Figure 6);

4. ***HIV Structure BLAST ***– Similarity search for analyzing HIV protein sequences with corresponding structural data (Figure 7);

5. ***Proteomics Online Tools ***– Directory of data resources and tools available for both protein sequence and protein structure analyses of HIV (Figure 8 &9).

## The proteome link

**Figure 10:**
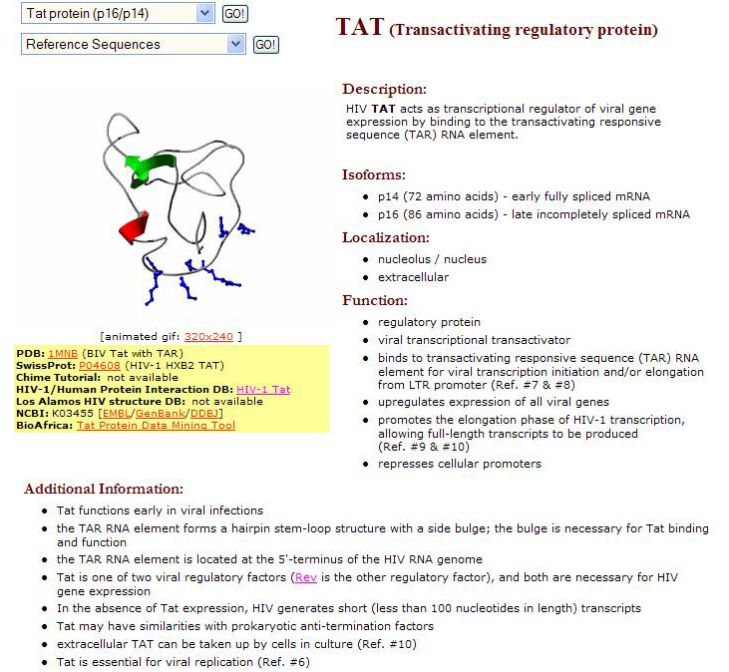
A general overview of the HIV-1 Proteome section of the BioAfrica website, as exemplified by the Tat web page .

In the HIV-1 Proteome section, each of the 19 HIV-1 proteins has a webpage that is divided into six parts: "general overview", "genomic location", "domains/folds/motifs", "protein-macromolecule interactions", "primary and secondary database entries", and "references and recommended readings" (Figure 4). The overview includes a description of the protein, a list of known isoforms, a representative tertiary structure animated image (GIF format) of the protein and its co-ordinates (PDB format), a link to chime tutorials, if available, and information about cleavage sites, localization, and functional activity. The genomic location section provides information on the location of the sequence relative to the reference sequence, HIV-1_HXB2 _[4], sequence data (fasta format), and information about the length, molecular weight and theoretical isoelectric point (pI) of the protein. The domains/folds/motifs section contains information about functional domains and predicted motifs (glycosylation, myristoylation, amidation, phosphorylation and cell attachment sites) of HIV-1_HXB2 _[4], and provides structural predictions (secondary structure, transmembrane regions, low complexity regions, and coiled-coil regions). The section on protein-macromolecule interactions includes information on protein complexes, protein-protein/DNA/RNA interactions, signal-transduction pathways, and potential interactions with other pathogens. The section on primary and secondary databases contains a list of database entries that are needed to retrieve information on protein structure, nucleotide/amino acid sequence data, protein sequence annotation, proteins with similar sequence and structure (such as Los Alamos National Laboratories HIV Sequence Database and the RCSB Protein Data Bank), as well as information on post-translational modification and protein-protein interactions. A list of key reviews and publications, used in the development of the BioAfrica HIV-1 Proteomics Resource, is provided in the references and recommended readings section. As an example, the proteome webpage for Tat, describes how this protein up-regulates HIV-1 gene expression by interacting with the long-terminal repeat (LTR) of HIV-1, promoting the elongation phase of viral transcription, allowing full-length HIV-1 mRNA transcripts to be produced [5,6] (Figure 10). The webpage also gives information on the structural organization of *tat *gene. The mRNA is derived from spliced exons encoded in two different open reading frames. In HIV-1_HXB2_, these reading frames are separated by a distance of 2334 nucleotides. Some HIV-1 isolates, including HIV-1_HXB2_, contain an artifact of laboratory strains consisting of a premature stop codon at position 8424 of exon 2. The presence of this stop codon leads to the synthesis of a truncated form of Tat that is 86, rather than 101 amino acids in length. The protein has two different isoforms – one translated from early-stage multiply spliced mRNA (p14); the other from singly-spliced mRNA (p16) [7]. Important functional domains include the acidic, amphipathic region (1-**M***E***PV***D***P***R***L***E***PW***KH***P**G*SQ***P***KT***A**-21; the hydrophobic residues are highlighted in bold, and polar residues are italicized) at the N-terminus of the protein; the cysteine-rich disulphide bond region (22-**C**TN**C**Y**C**KK**CC**FH**C**QV**C**-37); the core, basic and glutamine-rich region (49-**R**KK**RRQRRR**AH**Q**NS**Q**TH**Q**ASLSK**Q**-72) that is important for nuclear localization and TAR-binding activity, and the RGD cell-attachment site that binds to cellular integrins. In addition to being expressed in HIV-1-infected cells, Tat is also released into the extracellular fluid where it acts as a growth factor for the development of Kaposi's Sarcoma. Additional information about Tat and its protein-protein interactions can be found on the proteome page of the BioAfrica website located at .

## Protease cleavage sites link

Post-translational cleavage of the Gag, Gag-Pol and Nef precursor proteins occurs at the cell membrane during virion packaging, and is essential to the production of infectious viral particles. Drugs that inhibit this process, the protease inhibitors (PIs), are the most potent antiretroviral agents currently available. Thus it is important to collect information, not only on the sequence of protease enzymes from different HIV-1 subtypes, but also on the natural polymorphisms and resistance mutations that may effect their catalytic activities, drug responsiveness, substrate specificities, and cleavage site characteristics. Studies have shown that resistance mutations in the protease of subtype B are associated with impaired proteolytic processing and decreased enzymatic activity, and that compensatory mutations at Gag and Gag-Pol cleavage sites can partially overcome these defects [8]. These findings suggest that variation at protease cleavage sites may play an important role, not only in regulation of the viral life cycle, but also in disease progression and response to therapy.

The cleavage site section of the BioAfrica webpage is the direct extension of a recent publication in the Journal of Virology describing the location and variability of protease cleavage sites [9] (Figure 5). Together, these two resources provide information on the structure, amino acid composition, genetic variation and evolutionary history of protease cleavage sites, and on the natural selection pressures exerted on these sites. The section also serves as a baseline for understanding the impact of natural polymorphisms and resistance mutations on the catalytic efficiency of the protease enzyme, and on its ability to recognize and cleave individual Gag, Gag-Pol and Nef substrates. Such studies are important for understanding the mechanisms underlying the emergence of PI-induced drug resistance, and for designing alternative, optimized therapies.

## Protein data-mining tools link

The HIV-1 Protein Data-Mining Tool contains twelve sequence analysis techniques for assessing protein variability among different strains of HIV-1 (Figure 6). These tools allow the user to manipulate, analyze and compare published [9-12] and newly-acquired data in a user-friendly, hands-on manner. The analysis is initiated by selecting a particular subset of HIV-1 proteins, either from the user's database, or from the representative dataset of group M viruses (subtypes A through K). Using this dataset, the investigator can then perform a variety of protein-specific analyses. With a single click of the mouse, users can download the amino acid sequence in fasta format; obtain sequence annotations from SwissProt [13] or GenBank [14]; identify functional motifs using BLOCKS [15], PROSITE [16] or ProDom [17]; perform similarity searches using the BLAST program available at Genbank [18], conduct structural comparisons using the BioAfrica BLAST Structure program; determine amino acid composition, predict hydrophobicity and tertiary structure using the Swiss-Model homology modelling server [19], and obtain a list of potential protein-macromolecule interactions from the Database of Interacting Proteins (DIP) [20]. A representative analysis of HIV-1 Tat is shown in Additional file 1. The selected dataset, consisting of eight reference strains – four subtype B (HXB2-1983-France, RF-1983-US, JRFL-1986-US, WEAU160-1990-US) and four subtype C (92BR025-1992-Brazil, 96BW0502-1996-Botswana, TV002c12-1998-SouthAfrica, TV001c8.5-1998-SouthAfrica) isolates – were analyzed using PROSITE [16]. As shown in Additional file 1, all eight isolates had identical amidation, cysteine-rich and myristylation motifs at amino acid codons 47–50, 22–37 and 44–49, respectively. Three (75%) of the B isolates contained a second myristylation site at codons 42–47, as did three (75%) subtype C viruses. One (25%) of the C viruses carried an extra GNptGS myristylation motif at position 79–84. In addition, all four (100%) C isolates contained a novel myristylation motif, GSeeSK, at amino acid position 83–88, that was not present in four B viruses selected for study. However, the most striking difference between the two subtypes was the increased number of phosphorylation motifs in subtype C relative to B viruses. This increase, which occurs in cAMP/cGMP-dependent kinase, protein kinase C (PKC) and casein kinase II (CKII) phosphorylation sites, has been reported previously [21], but the significance of these findings remain to be established. The analysis also highlighted the atypical nature of the HIV-1_HXB2 _isolate, which, in addition to a premature stop codon, contained no cAMP/cGMP, PKC or CKII phosphorylation sites.

## The blast structure tool link

The HIV-1 BLAST Structure Tool facilitates the analysis of HIV-1 protein structure by allowing for rapid retrieval of archived structural data stored in the public databases (Figure 7). Users may input any HIV-1 amino acid sequence and obtain a list of similar HIV protein sequences for which structural data have been experimentally determined and deposited into the Protein Data Bank (PDB) [22]. After downloading the data from the PDB, subsequent structural analyses can be performed using the software programs and web-servers listed in the Proteomics Tools Directory. For example, a query using an amino acid sequence of HIV-1 Integrase protein from NCBI (gi|15553624|gb|AAL01959.1) results in a list of 54 structural models (ie. PDB_ID|1K6Y) within the PDB. Each of these structural models can be retrieved from the PDB, and the most appropriate structural model could be used for generating a homology model using the query protein sequence.

## The proteomics tools directory link

The HIV-1 Proteomics Tools Directory is divided into two web pages. The initial webpage is a concise compilation of some of the most commonly used protein-specific Internet resources (Figure 8). This "beginners" page displays a short list of websites for each of the following twelve categories: "protein databases", "specialized viral-protein databases", "motif and transcription factor databases", "protein sequence similarity searches", "protein sequence alignment", "protein sequence prediction tools", "protein sequence analysis", "protein sequence manipulation", "protein structure analysis", "molecular modelling tools", "tutorials", and "downloads". In addition, the Proteomics Tools Directory has an advanced web page for users who are looking for alternative, or more specialized, protein analysis tools (Figure 9). The advanced webpage displays a list of more than 200 links to different websites and web-servers. These data sources contain a variety of information ranging from specialized protein sequence databases to software programs capable of performing rigid body protein-protein molecular docking simulations.

## Conclusion

The impending rollout of antiretroviral therapy to millions of HIV-1-infected people in sub-Saharan Africa provides a unique opportunity to monitor the efficacy of non-B treatment programs from their very inception, and to obtain critical new information for the optimization of treatment strategies that are safe, affordable and appropriate for the developing world. An integral part of this massive humanitarian effort will be the collection of large amounts of clinical and laboratory data, including genetic information on viral subtype and resistance mutations, as well as routine CD4+ T-cell counts and viral load measurements. The mere collection of this data, however, does not ensure that it will be used to its maximum potential. To achieve full benefit from this explosive source of new information, the data will need to be appropriately collated, stored, analyzed and interpreted.

The rapidly emerging field of Bioinformatics has the capacity to greatly enhance treatment (and vaccine) efforts by serving as a bridge between Medical Informatics and Experimental Science. By correlating genetic variation and potential changes in protein structure with clinical risk factors, disease presentation, and differential response to treatment and vaccine candidates, it may be possible to obtain valuable new insights that can be used to support and guide rationale decision-making, both at the clinical and public health levels. The HIV-1 Proteomics Resource, described in this report, is an initial first step in the development of improved methods for extracting and analyzing genomics data, converting it into biologically useful information related to the structure, function and physiology of HIV-1 proteins, and for assessing the role these proteins play in disease progression and response to therapy. The Resource, developed at the Molecular Virology and Bioinformatics Unit of the Africa Centre of Health and Population Studies, is a centralized user-friendly database that is easily accessed through the BioAfrica website at [23].

## List of abbreviations used

AA – Amino Acid

BLAST – Basic Local Alignment Search Tool

CKII – casein kinase II

CTLs – cytotoxic T-lymphocytes

DIP – Database of Interacting Proteins

DNA – deoxyribonucleic acid

Env – envelope glycoprotein

Gag – group-specific antigen polyprotein

GIF – Graphics Interchange Format

HIV – Human Immunodeficiency Virus

HIV-1 – Human Immunodeficiency Virus Type-1

HTTP – Hypertext Transfer Protocol

LTR – long-terminal repeat

mRNA – messenger RNA

NCBI – National Center for Biotechnology Information

Nef – negative factor

PDB – Protein Data Bank

pI – isoelectric point

PIs – protease inhibitors

PKC – protein kinase C

Pol – polymerase polyprotein

Rev – ART/TRS anti-repression transactivator protein

RNA – ribonucleic acid

RNase H – ribonuclease H

Tat – transactivating regulatory protein

Vif – virion infectivity factor

Vpr – viral protein R

Vpu – viral protein U

## Competing interests

The author(s) declare that they have no competing interests.

## Authors' contributions

RSD created and maintains BioAfrica's HIV proteomics resource, HIV proteome section, proteomics tools directory, HIV-1 protein data-mining tool and HIV structure BLAST tool; performed protein sequence and structural model analyses; and wrote the manuscript.

TDO conceived and maintains the BioAfrica website, and continues to oversee its rapid expansion; created the cleavage sites section; and participated in the design and implementation of the HIV proteomics resource.

CS participated in the design of the HIV proteomics resource, with an emphasis on the proteomics tools directory.

SD participated in the design and creation of the HIV proteome section, with an emphasis on the HIV-1 Tat protein.

MG participated in the design of the HIV proteomics resource, with an emphasis on the HIV proteome section.

SC supervised the project, and participated in the design and implementation of the HIV proteomics resource.

All authors read and approved the final manuscript.

## Supplementary Material

Additional File 1 A table containing a comparative summary of potential functional motifs (cysteine-rich region, myristoylated Asparagine, amidation, cAMP- and cGMP- dependent kinase phosphorylation, Protein Kinase C phosphorylation, and Casein Kinase II phosphorylation) in the HIV-1 Tat proteins of subtypes B and C, as identified using PROSITE. Click here for file
