# Supplementary figures and images for: BioAfrica's HIV-1 Proteomics Resource: Combining protein data with bioinformatics tools
Source: Retrovirology. 2005 Mar 9;2:18. doi: 10.1186/1742-4690-2-18 (PMC555852; doi:10.1186/1742-4690-2-18)

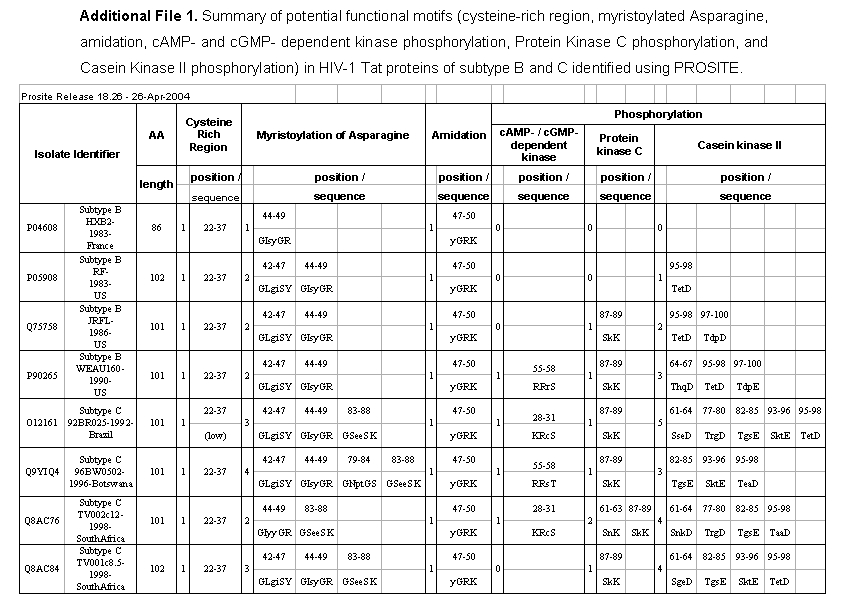

Supplement: Additional File 1 — A table containing a comparative summary of potential functional motifs (cysteine-rich region, myristoylated Asparagine, amidation, cAMP- and cGMP- dependent kinase phosphorylation, Protein Kinase C phosphorylation, and Casein Kinase II phosphorylation) in the HIV-1 Tat proteins of subtypes B and C, as identified using PROSITE. [file 1742-4690-2-18-S1.jpeg]
